# Dipeptidyl Peptidase-4 Inhibitor Decreases Abdominal Aortic Aneurysm Formation through GLP-1-Dependent Monocytic Activity in Mice

**DOI:** 10.1371/journal.pone.0121077

**Published:** 2015-04-14

**Authors:** Hsin Ying Lu, Chun Yao Huang, Chun Ming Shih, Wei Hung Chang, Chein Sung Tsai, Feng Yen Lin, Chun Che Shih

**Affiliations:** 1 Institute of Clinical Medicine, National Yang-Ming University, Taipei, Taiwan; 2 Department of Internal Medicine, School of Medicine, College of Medicine, Taipei Medical University, Taipei, Taiwan; 3 Division of Cardiology, Department of Internal Medicine, Taipei Medical University Hospital, Taipei, Taiwan; 4 Division of Cardiovascular Surgery, Tri-Service General Hospital, Taipei, Taiwan; 5 Division of Cardiovascular Surgery, Taipei Veterans General Hospital, Taipei, Taiwan; Max-Delbrück Center for Molecular Medicine (MDC), GERMANY

## Abstract

Abdominal aortic aneurysm (AAA) is a life-threatening situation affecting almost 10% of elders. There has been no effective medication for AAA other than surgical intervention. Dipeptidyl peptidase-4 (DPP-4) inhibitors have been shown to have a protective effect on cardiovascular disease. Whether DPP-4 inhibitors may be beneficial in the treatment of AAA is unclear. We investigated the effects of DPP-4 inhibitor sitagliptin on the angiotensin II (Ang II)-infused AAA formation in apoE-deficient (apoE^-/-^) mice. Mice with induced AAA were treated with placebo or 2.5, 5 or 10 mg/kg/day sitagliptin. Ang II-infused apoE^-/-^ mice exhibited a 55.6% incidence of AAA formation, but treatment with sitagliptin decreased AAA formation. Specifically, administered sitagliptin in Ang II-infused mice exhibited decreased expansion of the suprarenal aorta, reduced elastin lamina degradation of the aorta, and diminished vascular inflammation by macrophage infiltration. Treatment with sitagliptin decreased gelatinolytic activity and apoptotic cells in aorta tissues. Sitaglipitn, additionally, was associated with increased levels of plasma active glucagon-like peptide-1 (GLP-1). *In vitro* studies, GLP-1 decreased reactive oxygen species (ROS) production, cell migration, and MMP-2 as well as MMP-9 activity in Ang II-stimulated monocytic cells. The results conclude that oral administration of sitagliptin can prevent abdominal aortic aneurysm formation in Ang II-infused apoE^-/-^mice, at least in part, by increasing of GLP-1 activity, decreasing MMP-2 and MMP-9 production from macrophage infiltration. The results indicate that sitagliptin may have therapeutic potential in preventing the development of AAA.

## Introduction

Surgical intervention is currently the only therapy accessible for the development of abdominal aortic aneurysms (AAAs). Upon the insufficient-effective medical treatment to date, AAA rupture remains a significant cause of mortality in the elderly. The status is further complicated by the fact that surgical intervention in and of itself is associated with a 10% risk of death [1]. Under the increase in the aged prevalence in AAA, novel medical treatments intend reducing the progression of AAA have been expected. Still unclear the etiology of AAAs, the current literature suggests that elastin and extracellular matrix (ECM) degradation play a key role in the pathogenesis of the condition [2]. Reportedly having been indicated that chronic vascular inflammation is a hallmark of AAA. The main source of proteases from the infiltrated monocytes and macrophages into the vessel wall destroy the integrity of the aortic wall and degrade ECM, thus contributing to the development, progression and rupture of AAA [3]. The process of vascular inflammation involves an "inside-out" response that arises from endothelial activation and leukocyte extravasation that proceed toward the adventitia, but a "outside-in" hypothesis has come into view in AAA formation whereby vascular wall inflammation is initiated in the adventitial layer and progresses through the media toward the intima instead [4].

Dipeptidyl peptidase-4 (DPP-4), also known as lymphocyte cell surface marker CD26, exists both as membrane-anchored cell-surface peptidase and as a smaller soluble form in blood plasma. DPP-4 is widely expressed on T cells and B cells, natural killer cells, subsets of macrophages, hematopoietic stem cells, and hematopoietic progenitor cells, as well as on epithelial, endothelial, and acinar cells of a variety of tissues including [5,6]. A soluble form of DPP-4 that lacks intracellular and transmembrane regions presents in body fluids such as urine, serum/plasma, seminal plasma and amniotic fluid, yet the origin of soluble DPP-4 are not completely understood. The complex biological roles of DPP-4 include cell membrane associated activation of intracellular signal transduction pathways, cell-to-cell interaction, and enzymatic activity [7]. Inhibition of the DPP-4 system trends a new approach in the management of Type-2 diabetes by virtue of its effects on extending the half-life of glucose-dependent insulinotropic peptide (GLP-1) and glucagon-like peptide-1 (GIP) [8]. DPP-4 inhibitor has been demonstrated to play a protective role in cardiovascular diseases, including hypertension [9], cardiomyopathy [10], atherosclerosis [11], and peripheral vascular disease [12] via both GLP-1 dependent and independent effects. However, there is a lack of evidence supporting a beneficial effect of DPP-4 inhibitors on AAA. Recently, one investigator reported that the DPP-4 inhibitor ameliorated AAA by inhibiting oxidant stress [13]. Nevertheless, the mechanism underlying the effects of DPP-4 inhibition is unclear. Thus, the purpose of this study was to evaluate the role of DPP-4 inhibitor in experimental AAA pathogenesis, with the extreme ambition of identifying novel medication for the treatment of AAAs.

## Materials and Methods

### Animal preparation and drugs administration

Male apoE^-/-^ mice on a C57BL/6 background were purchased from Jackson Laboratories (Bar Harbor, ME, USA). Mice were kept in micro-isolator cages on a 12-h day/night cycle. Water and a normal laboratory diet were available *ad libitum*. All experiments were performed in 12- to 14-week-old male apoE^-/-^ mice. Mice were infused subcutaneously with Ang II (1000 ng/kg/min; Sigma, St. Louis, MO, USA) or normal saline (NS) via mini-osmotic pumps (model 2004, Alzet, Palo Alto, CA, USA) for 28 days [14]. The mice were divided into 5 groups and, orally administered the equally volume of water as vehicle or the DPP-4 inhibitor, sitagliptin (Januvia, Merck & Company, Inc., Rahway, NJ, USA) at the following doses: 0 mg/kg/day; 2.5 mg/kg/day; 5 mg/kg/day; 10 mg/kg/day for 28 days after pump implantation. The doses used in mice were comparable to doses given to human on the basis of body surface. Also, mice were treated with liraglutide (Victoza, Novo Nordisk, Inc., Plainsboro, NJ, USA) at a modify previously used dosed of s.c. 400 μg/kg daily twice for 28 days after pump implantation [15]. At the end of the study, mice were euthanized by exsanguination under anesthesia (ketamine-HCl 100 mg/kg and xylazine 20 mg/kg given i.p., animals were considered as adequately anaesthetized when no attempt to withdraw the limb after pressure could be observed). All experimental protocols and procedures were approved by the Institutional Animal Care and Use Committee of the National Yang-Ming University (Taipei, Taiwan).

### Measurement of blood pressure

Blood pressure was measured in conscious mice using a noninvasive tail-cuff system (Softron, BP-98A). All mice were trained to stay quietly in a restrainer that was placed on a warm pad for a period of at last 30 minutes before the measurements. Blood pressure was measured repeatedly and recorded. Mean arterial pressure (MAP) was calculated. MAP was averaged from 3 consecutive measurements.

### Biochemical measurements

Blood samples for biochemical measurements were collected from each animal before and at the end of the experiment. Samples were obtained from the mandibular artery without sedation, collected into EDTA-containing tubes and separated by centrifugation at 1000xg for 10 min at 4°C. Plasma samples were stored at -80°C until tested. Plasma total cholesterol, triglyceride, high-density lipoprotein (HDL) and low-density lipoprotein (LDL) were measured using a SPOTCHEMTM automatic dry chemistry system (SP-4410; Arkray, Shanghai, Japan). ELISAs were performed to determine the plasma level of SDF-1α (R&D Systems Inc., Minneapolis, MN, USA), DPP-4 (RayBiotech Inc., Norcross, GA, USA), DPP-4 activity (Enzo Life Sciences, Lausen, Switzerland), and GLP-1 activity (Millipore, Billerica, MA, USA).

### Characteristics and quantification of AAA

After the aorta was dissected free from the surrounding connective tissue, images were captured with a digital camera and used to measure the outer diameter of the suprarenal aorta at the midpoint between the diaphragm and right renal artery. An abdominal aortic aneurysm was defined as a 50% increase in external diameter of the abdominal aorta [14,16]. The lumen and adventitial circumferences at the maximally expanded portion of the suprarenal aorta were quantified using Image J software (National Institutes of Health, Bethesda, MD, USA). All measurements were performed by the same operator to avoid inter-observer variability.

### Histological and immunofluorescent staining

4 μm thickness of aortic cross-sections were analyzed by hematoxylin/eosin (H&E) staining Verhoeff-Van Giessen (VvG) for elastin, masson’s trichrome for the assessment collagen and F4/80 (AbD seroTec, San Francisco, CA, USA) for macrophages. To identify macrophages and MMPs expression in the aortic wall, an immunohistochemical assay was performed. The paraffin-embedded sections were incubated with the antibodies anti-MMP-2 (Millipore, Billerica, MA, USA), anti-MMP-9 (Abcam, Cambridge, MA, USA), anti-TIMP-1 and anti-TIMP-2 (Bioss, Woburn, MA, USA).

### TUNEL assay

To localize cells undergoing nuclear DNA fragmentation, terminal deoxynucleotidyl transferase dUTP nick end labeling (TUNEL) was performed using in a situ apoptosis detection kit (Roche, Branchburg, NJ, USA) according to the manufacturer’s protocol. Briefly, paraffin sections were deparaffinized and rehydrated. Sections were then washed with PBS and incubated with proteinase K (20 mg/ml) for 40 minutes. TdT, which catalyzes a template-independent addition of deoxyribonucleotide to 3-OH ends of DNA, was used to incorporate digoxigeninconjugated dUTP to the ends of DNA fragments in situ. The TUNEL signal was then detected with an anti-digoxigenin antibody conjugated with peroxidase and developed with diaminobenzidine as a chromogen. Nuclei were counterstained with DAPI. Images were obtained using a Zeiss LSM 510 Meta confocal microscope (Carl Zeiss MicroImaging Inc. USA).

### Gelatin zymography

Gelatin zymography was used to determine the gelatinolytic activity of MMP-2 and MMP-9 in homogenates of aortas and in cell culture supernatant as previously described. In vivo experiment, AAA samples were pooled into 1 set of 3 samples and analyzed. Experiments were performed in triplicate, and 1 representative experiment was shown. Briefly, equivalent amounts of sample were electrophoresed under non-reducing conditions on 7.5% SDS-PAGE gels containing 0.1 mg/mL gelatin as a substrate. The gels were washed in a buffer containing 2.5% Triton X-100 for 1 h to remove SDS, and then incubated with a substrate buffer at 37°C for 18 h. MMPs activity was quantified by densitometry scanning. The data obtained from densitometric analysis were expressed as fold-change in activity relative to controls.

### Cell culture

Primary cultures of human aortic adventitial fibroblast (hAoF) (Cambrex- Lonza, Walkersville, Md., USA) were purchased, grown in stromal cell growth medium (SCGM) containing 5% FBS, and used from passages 4 to 9. These cells were obtained from normal aortic tissue and their identity and purity were confirmed based on morphology, growth characteristics, and negative immunofluorescent staining for smooth muscle alpha-actin. Monocytic U937 cells were obtained from American Type Culture Collect. U937 cells were maintained in RPMI 1640 culture medium supplemented with 10% heat-inactivated FCS, 1% glutamine and 1% penicillin-streptomycin at 37°C in a humidified atmosphere containing 5% CO2.

### Measurement of intracellular generation of reactive oxygen species (ROS)

ROS were measured using a previously described method. In brief, U937 cells were pre-treated with GLP-1 for 1 hour and then treated with Ang II (1 μM) for 24 hours. Cells were loaded with DCFH-DA (5 μM) (Invitrogen Corp.,Carlsbad, CA, USA) for 30 min at 37°C. ROS level could be monitored at 488 nm excitation and 515 nm emission.

### Macrophage chemotaxis assay

The migration of U937 monocyte to hAoF-conditioned medium was assessed using transwell polycarbonate membrane inserts with 5 μm pores (Millipore, Billerica, MA, USA). Briefly 2x10^5^ U937 cells in 100 ml of serum free SCGM were placed above the inserts, and 600 ml of hAoF conditioned medium or SCGM was placed below. Cells were treated with liraglutide at a concentration of 10, 50, or 100 nM for 1 hour. Ang II (1 μM) was then added to stimulate cell migration. After incubation for 4 h at 37°C, filters were fixed and stained, and cells that had migrated through the filter were counted under a high power field. For each group, 5 high power images were used and all experiments were repeated three times.

### Statistical analysis

Values are expressed as the mean ± SEM. Statistical evaluation was performed using Student’s t-test and one- or two-way ANOVA, followed by Dunnett’s. A probability value of P < 0.05 was considered significant.

## Results

### Sitagliptin did not affect biochemical parameters except for mean arterial pressure in Ang II-induced apoE^-/-^ mice

To assess the influence of sitagliptin on the characteristics of Ang II-infused AAA in apoE^-/-^ mice, body weight, mean arterial pressure (MAP) and biochemical measurements were taken. As shown in Table 1, treatment with sitagliptin in Ang II-infused apoE^-/-^ mice did not affect body weight, lipid profiles and glucose. Because hypertension is induced by Ang II, we also examined this parameter in the control and treated apoE^-/-^ mice. Ang II infusion significantly increased MAP in apoE^-/-^ compared with NS-infused mice (134.1 ± 8.9 mmHg and 101.3 ± 3.6 mmHg, respectively). Treatment with 2.5, 5 and 10 mg/kg/day of sitagliptin in Ang II-infused mice significantly decreased MAP (111.6 ± 8.6 mmHg, 108.9 ± 6.8 mmHg and 113.7 ± 6.4 mmHg, respectively). The results showed that administration of sitagliptin did not affect the lipid profile, but it did decrease MAP in Ang II-induced apoE^-/-^ mice.

**Table 1:** Effects of daily sitagliptin and Ang II on physiological and biochemical characteristics.

Sitagliptin	NS	administration of Ang II			
	0 (mg/kg/day) n = 10	0 (mg/kg/day) n = 10	2.5 (mg/kg/day) n = 12	5 (mg/kg/day) n = 8	10 (mg/kg/day) n = 10
BW (g)	32.4 ±3.3	31.0 ± 1.1	29.5 ± 2.4	29.6 ± 2.5	28.3 ±2.1^#^
MAP (mmHg)	101.3 ± 3.6	134.1 ± 8.9*	111.6 ± 8.6^#^	108.9 ± 6.8^#^	113.7 ± 6.4^#^
Pulse rates (beats/min)	668.3 ± 19.1	683.0 ± 24.6	679.2 ±30.1	675.0 ± 47.6	684.2 ± 33.3
t-Cho (mg/dL)	595.1 ±98.5	526.3 ± 91.9	490.7 ± 68.1	504.2 ± 49.5	488.3 ± 58.4
TG (mg/dL)	82.3 ± 15.9	78.3 ± 30.8	65.6 ± 29.3	92.2 ± 52.5	93.8 ± 39.1
HDL-c (mg/dL)	82.9 ± 23.2	87.2 ± 36.7	65.5 ± 10.7	69.0 ± 13.4	73.5 ± 13.4
LDL-c (mg/dL)	465.8 ± 76.3	390.6 ± 49.5	412.0 ± 57.3	419.6 ± 13.4	393.8 ± 61.6
Glucose (mg/dL)	139.2 ± 2.9	142.8 ± 2.3	137.5 ± 3.8	134.3 ± 3.1	131.0 ± 6.2

NS: Normal saline; BW: body weight; MAP: mean arterial pressure; t-Cho: total cholesterol; TG: triglycerides; HDL: High-density lipoprotein; LDL: Low-density lipoprotein. Data are expressed by mean±SEM.

*P<0.05, compared with mice of NS infusion.

^#^ *P*<0.05, compared with mice of Ang II infusion with 0 mg/kg/day sitagliptin administration.

### Sitagliptin attenuates Ang II-induced AAA in apoE^-/-^ mice

**Fig 1:**
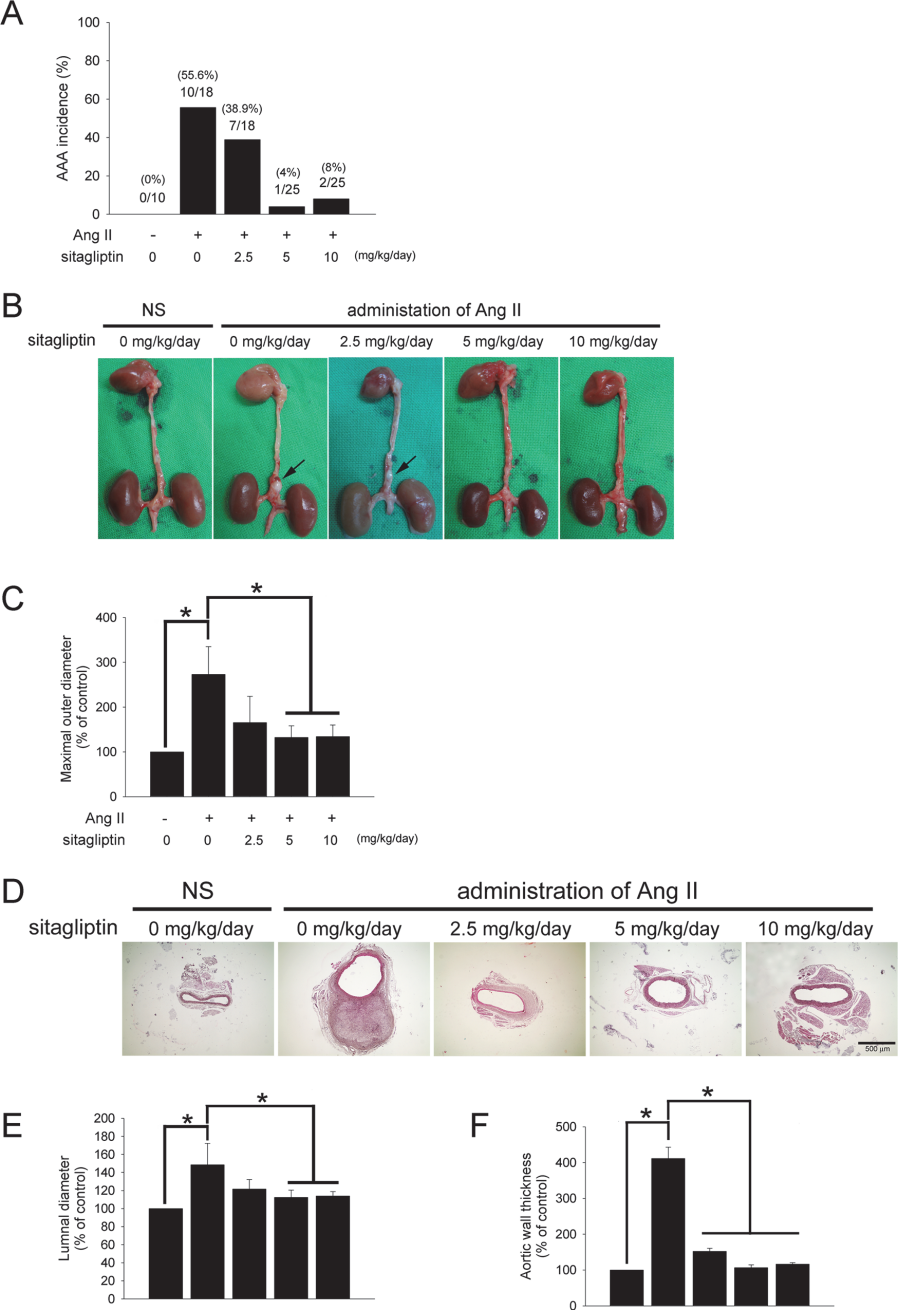
Sitagliptin attenuated AAA formation in Ang II-infused apoE^-/-^ mice. (A) Histogram representing the percent incidence of AAAs for each treatment group. (n = 10–25 per group) (B) Representative photographs showing the features of aneurysms induced by Ang II. The arrow indicates typical AAAs. (C) Quantification of the maximal outer diameters of the suprarenal aortas in apoE^-/-^ mice infused with NS or Ang II given as percentages. (*P < 0.05, n = 10 per group) (D) Representative staining with H&E of the suprarenal aortas of mice. The magnification of images shown is 40x. (n = 5 per group) (E and F) Quantification of the luminal diameters and wall thickness of suprarenal aortas in apoE^-/-^ mice infused with NS or Ang II given as percentages. (*P < 0.05, n = 5 per group).

To determine the role of sitagliptin in Ang II-induced AAAs, sitagliptin was administered to male apoE^-/-^ mice given saline or Ang II infusion. Following the 28 days of infusion, mice were euthanized and aortae were isolated and examined for the presence of AAA. In a previous report, aneurysms were noted to localize to the suprarenal portion of the abdominal aorta with an incidence of 60% [17]. In our study, the total incidence of AAAs (excluding AAA-associated deaths) in the suprarenal aorta of Ang II-infused mice was 55.6%. No spontaneous AAAs were observed in the NS-infused mice. Co-infusion of Ang II with different doses of sitagliptin strikingly reduced AAA incidence, with only 4% occurring in the 5 mg/kg/day and 8% in 10 mg/kg/day sitagliptin-treated mice (Fig 1A).

After the mice were euthanized, the outer diameter of the aorta was assessed ex vivo by measuring the maximal width of the dissected suprarenal aorta and quantified from the image captured by a calibrated digital camera via image J software. As shown in Fig 1C, Ang II infusion significantly increased the maximal outer diameter of the aorta compared with NS infusion mice (1.3 ± 0.0 mm and 3.6 ± 0.8 mm, respectively), but 5 and 10 mg/kg/day of sitagliptin significantly decreased the maximal outer diameter of the aorta (2.2 ± 0.8 mm and 1.7 ± 0.3 mm, respectively).

H&E staining was performed for histological examination (Fig 1D). The analysis of the maximal aortic and luminal diameters was shown in Fig 1E. Compared NS, Ang II infusion markedly increased the aortic lumen by 148.6 ± 23.8%, but mice treated with 5 and 10 mg/kg/day of sitagliptin had a significantly reduced lumen (112.4 ± 8.1% and 113.8 ± 5.0%, respectively). Moreover, Ang II-infusion increased the wall thickness (411.3 ± 31.6% of control), but mice treated with 2.5, 5, 10 mg/kg/day of sitagliptin had a significant reduction in aortic wall thickness (152.2 ± 8.3%, 106.7 ± 8.0%, 116.7 ± 3.8%, respectively) (Fig 1F). Our results indicated that sitagliptin prevents AAA formation in Ang II-infused apoE^-/-^ mice.

### Sitagliptin ameliorated Ang II-induced morphological changes in the aortic wall

**Fig 2:**
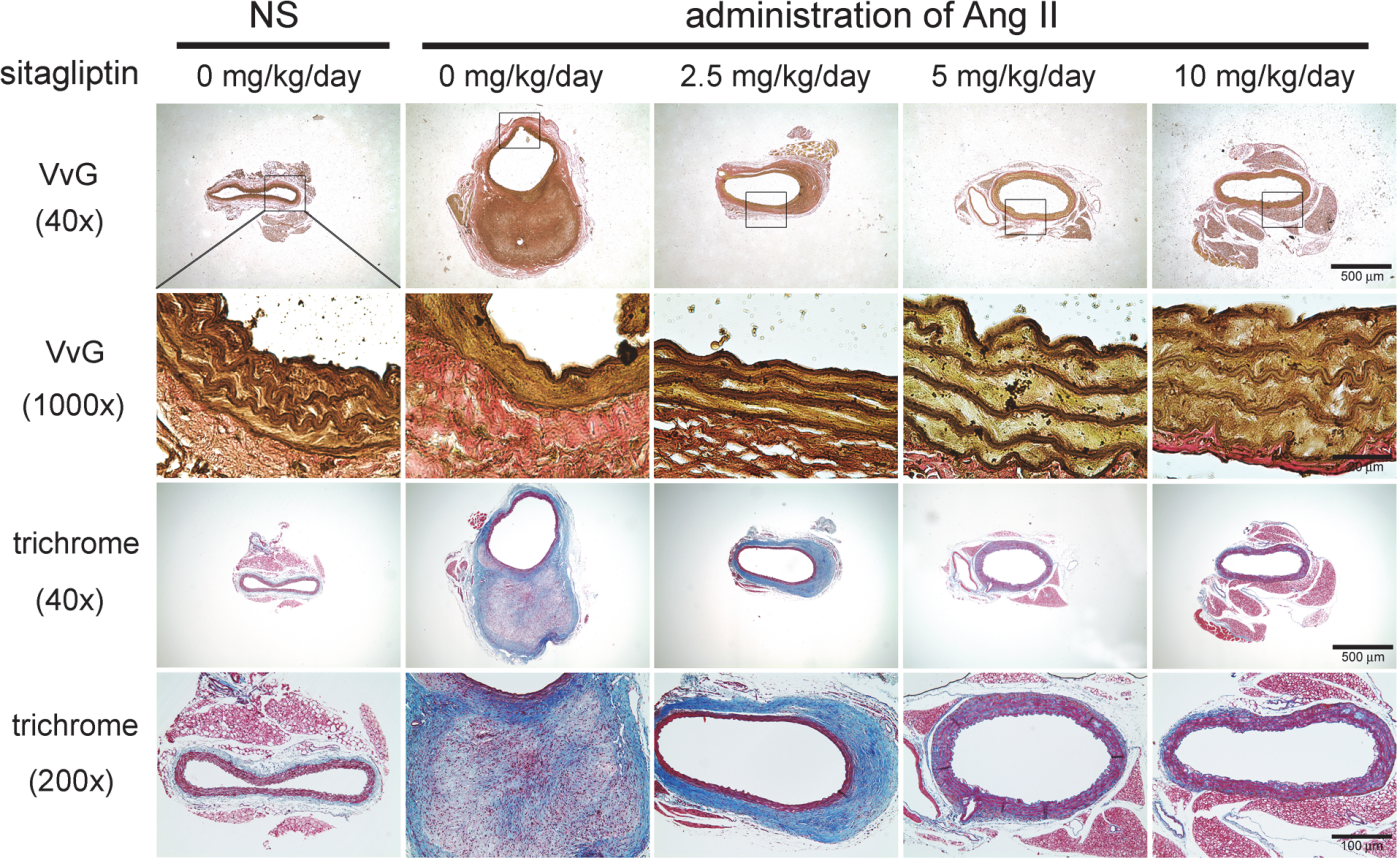
Sitagliptin ameliorated Ang II-induced morphological and biochemical changes in the aortic wall. Representative aortic sections of the suprarenal aorta stained with Verhoeff -Van Giessen (VvG) and Masson’s trichrome from apoE^-/-^ mice treated with NS or Ang II (n = 5 per group). Elastin staining in Ang II-infused mice revealed that the internal elastic lamina was discontinuous compared with NS-infused mice. Treatment with 2.5 mg/kg/day sitagliptin attenuated the rupture of the elastin laminas, but the elastic fibers still had less loose elastin elements. Administration of 5 and 10 mg/kg/day of sitagliptin significantly ameliorated the curve and density of the elastin laminas. Compared with NS-infused mice, trichrome staining of the aortas from Ang II-infused mice showed that medial smooth muscle and fibrous cells were markedly separated by stainable collagen. Treatment with 2.5 mg/kg/day of sitagliptin showed decreased adventitial matrix staining. Administration of 5 and 10 mg/kg/day of sitagliptin significantly prevented changes in the cellular components of the aortic wall. Masson's Trichrome stain, muscle is stained red, collagen—blue, fibrin—pink.

Collagen and elastin are the most abundant fibrous proteins of the arterial wall and are responsible for its characteristic mechanical resistance, tensile strength and elasticity [18]. Changes in their content and/or quality are likely to play a key role in the development of AAA. To investigate the effect of sitagliptin on the alterations of elastin and collagen, Verhoeff -Van Giessen (VvG) and Masson’s trichrome staining were performed. The results clearly showed that elastin was disrupted in Ang II-infused compared with NS-infused arteries (Fig 2). Indeed, the histology of the NS-infused arteries showed densely packed parallel elastin fibers uniformly arranged as opposed to the disrupted irregular pattern observed with the Ang II-infused arteries. However, treatment with 2.5 mg/kg/day of sitagliptin attenuated the rupture of elastin laminas, though the elastic fibers still had fewer loose elastin elements. Administration of 5 and 10 mg/kg/day of sitagliptin significantly ameliorated the curve and density of elastin laminas. Prior reports suggested that larger discontinuities were associated with replacement of the media and adventitia by fibrous connective tissue in Ang II-treated arteries. To exam whether sitagliptin alter the expression of cellular components by Ang II stimulation, Masson’s trichrome staining was performed. As shown in Fig 2, the aortas from the NS-infused mice had minimal collagen staining in the media and adventitia. The adventitial thickening of the aortic wall in Ang II-infused mice was also attributable to the deposition of extracellular fibers that surrounded smooth muscle cells. However, treatment with 2.5 mg/kg/day of sitagliptin not only decreased aortic wall thickness but also reduced fibrin formation. Treatment with 5 and 10 mg/kg/day of sitagliptin significantly decreased smooth muscle cell-rich area in Ang II-infused mice. Our results demonstrated that stagliptin protects structural integrity by preserving the elastin content as well as the distribution of cellular components.

### Sitagliptin reduces macrophages infiltration and gelatinase Activity in Ang II-infused apoE^-/-^ mice

**Fig 3:**
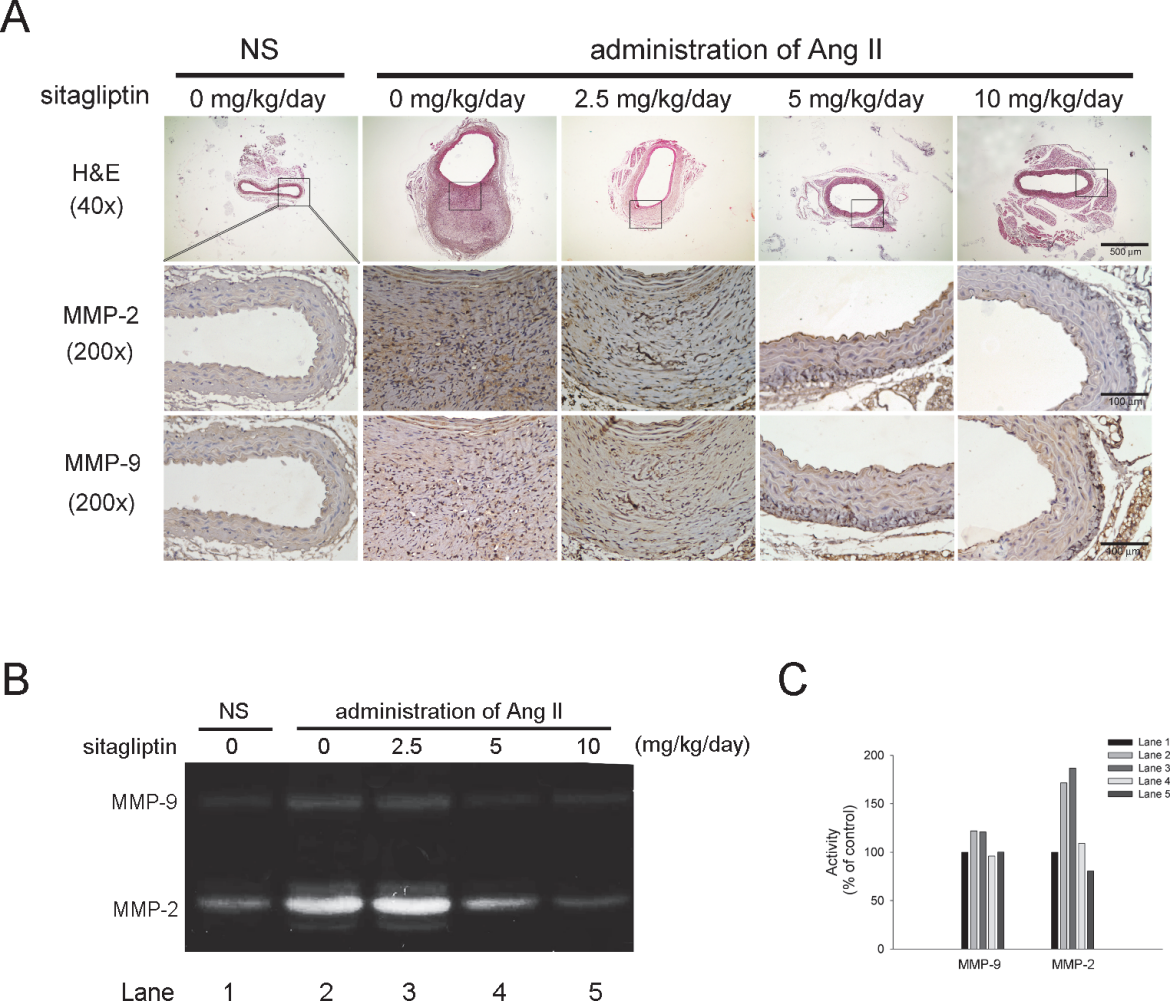
Sitagliptin suppressed macrophage infiltration and gelatinase activity in Ang II-infused apoE^-/-^ mice. (A) Representative immunostaining of F4/80, a specific marker of mature macrophages, MMP-2 and MMP-9. In the Ang II-infused aorta, the macrophage staining appeared to be much more prominent in the adventitia than in the media. Treatment with sitaglitin significantly decreased macrophages infiltration as well as MMP-2 and MMP-9 expression. The black arrow indicated macrophage and MMP-2 and MMP-9. The magnification of immunostaining images is 200x. (n = 5, per group) (B) Gelatin gel zymography to detect MMPs activity in aortic tissue extracts from different groups of mice. Aortic samples were pooled into 1 set of 3 samples each. (C) Quantitative densitometric analysis of MMP expression.

One mechanism by which oxidative stress contributes to AAA is attracting macrophages to elastin degradation products present in the aortic wall [19]. To evaluate the effect of sitagliptin on macrophage infiltration in AAA formation in this experimental model, immunostaining of F4/80, a specific marker of mature macrophages, was performed. The results showed that, compared with NS-infused apoE^-/-^ mice, Ang II infusion markedly increased macrophage infiltration, particularly in the adventitia layer, but in mice treated with 2.5,5 and 10 mg/kg/day of sitagliptin, there was significant reduction in the infiltration of macrophages (Fig 3A). A combination of matrix metalloproteinase (MMP), which has the ability to extensively degrade components of the ECM such as elastin, also played a critical role in the initiation and progression of AAA. To delineate the mechanism by which sitagliptin protects against aneurysmal development, we first examined the protein expression of MMP-2 and MMP-9 by IHC. As shown in Fig 3A, Ang II-infused apoE^-/-^ mice showed increased MMP-2 and MMP-9 expression compared with NS-infused aoE^-/-^ mice, however, treatment with 5 and 10 mg/kg/day of sitagliptin greatly decreased MMP-2 and MMP-9 expression. The arrows indicated macrophages, MMP-2 and MMP-9. Furthermore, we determined the gelatinolytic activity in the abdominal aortic tissue homogenates. Ang II-infused apoE^-/-^ mice exhibited significantly higher MMP-2 and MMP-9 activity, while apoE^-/-^ mice treated with 5 and 10 mg/kg/day sitagliptin showed lower activity (Fig 3B). The results showed that macrophages infiltration to aortic adventitia would be eliminated by sitagliptin as well as abolishing the coexistence of MMP-2 and MMP-9.

### Sitagliptin reduces aortic apoptosis in Ang II-infused apoE^-/-^ mice

**Fig 4:**
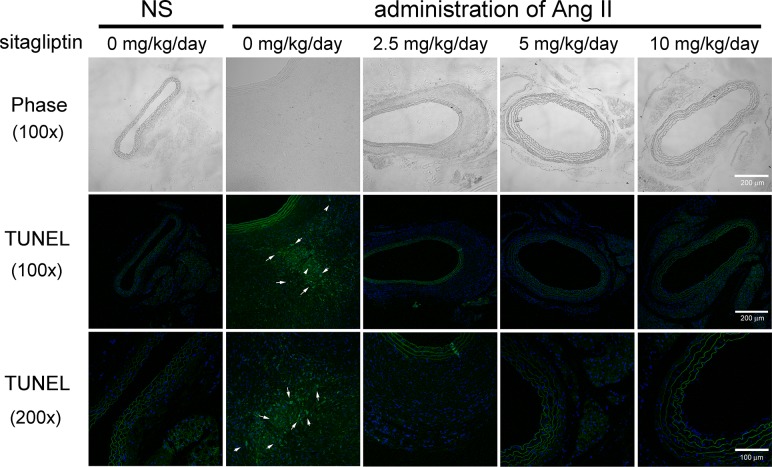
Treatment with sitagliptin suppresses apoptosis in Ang-infused apoE^-/-^ mice. Representative photographs of deoxynucleotidyl transferase dUTP nick end labeling (TUNEL) staining. Apoptotic cells are shown in green and the cell nuclei in blue. The white arrowheads indicate the apoptotic cells. Ang II significantly increased apoptotic cells, particularly in the adventitia layer, but sitagliptin greatly suppressed cell apoptosis (n = 5 per group). The images of the upper panel represent the visible light phase. The magnification of the middle and lower panels was 100x and 200x, respectively.

Apoptosis is an important pathological mechanism that alters tissue structure in AAA. To assess the effect of sitagliptin on apoptotic cells in the Ang II-infused aorta, we performed a TUNEL assay. As shown in Fig 4, a significant amount of apoptotic signal persisted in the Ang II-infused aorta, with most of the apoptotic cells localized in adventitia. This increase in apoptosis induced by Ang II was significantly ameliorated in the sitagliptin-treated mice. The result indicates that sitagliptin blockade of apoptosis may attenuate the pathological process that leads to the formation of AAA.

### Sitagliptin activates plasma active GLP-1 level

Prior evidence suggested that sitagliptin may regulate the production and activity of several proteins such as SDF-1, GLP-1, and DPP-4. Therefore, in this study, ELISAs were performed to monitor the plasma levels of SDF-1 and DPP-4, as well as the activity of DPP-4 and GLP-1. As shown in Table 2, plasma DPP-4 concentration significantly decreased in Ang II-infused apoE^-/-^ mice compared with NS-infused mice (292.8 ± 23.1 ng/mL and 577.5 ± 55.1 ng/mL, respectively). Treatment with 2.5, 5 and 10 mg/kg/day of sitagliptin significantly increased the plasma DPP-4 concentration (508.8 ± 74.8 ng/mL, 548.0 ± 42.9 ng/mL and 583.3 ± 90.4 ng/mL, respectively). However, increasing plasma DPP-4 activity was observed in Ang II-infused apoE^-/-^ mice compared with NS-infused apoE^-/-^ mice (142.8 ± 2.3 relative intensity and 139.2 ± 2.9 relative intensity, respectively). DPP-4 activity was greatly decreased in 2.5, 5 and 10 mg/kg/day sitagliptin-treated apoE^-/-^ mice (137.5 ± 3.8 relative intensity, 134.3 ± 3.1 relative intensity, 131.0 ± 6.2 relative intensity, respectively). Additionally, plasma active GLP-1 concentration was significantly decreased in the Ang II-infused apoE^-/-^ mice compared with NS-infused mice (0.8 ± 0.3 pM and 1.7 ± 0.4 pM, respectively). Treatment with 2.5, 5 and 10 mg/kg/day of stagliptin significantly increased the concentration of active GLP-1 in plasma (1.2 ± 0.3 pM, 1.4 ± 0.5 pM, and 1.2 ± 0.4 pM respectively). Ang II infusion did not affect plasma SDF-1α among groups. These observations suggest that sitagliptin may play a protective role via the promotion of plasma active GLP-1 level by inhibiting DPP-4 activity.

**Table 2:** The alternation of DPP-4 substrate by daily sitagliptin administration.

Sitagliptin	NS	administration of Ang II			
	0 (mg/kg/day) n = 10	0 (mg/kg/day) n = 10	2.5 (mg/kg/day) n = 10	5 (mg/kg/day) n = 9	10 (mg/kg/day) n = 8
SDF-1α (ng/mL)	1.3 ± 0.3	1.2 ± 0.2	1.2 ± 0.2	1.3 ± 0.3	1.2 ± 0.4
DPP-4 (ng/mL)	577.5 ± 55.1	292.8 ± 23.1*	508.8 ± 74.8^#^	548.0 ± 42.9^#^	583.3 ± 90.4^#^
DPP-4 activity (relative intensity)	139.2 ± 2.9	142.8 ± 2.3*	137.5 ± 3.8^#^	134.3 ± 3.1^#^	131.0 ± 6.2^#^
active GLP-1 (pM)	1.7 ± 0.4	0.8 ± 0.3*	1.2 ± 0.3^#^	1.4 ± 0.5^#^	1.2 ± 0.4^#^

NS: Normal saline; Data are means±SEM.

*P<0.05, compared with mice of NS infusion.

^*#*^ *P*<0.05, compared with mice of Ang II infusion with 0 mg/kg/day sitagliptin administrated.

### Liraglutide reduced AAA formation in response to elevated plasma of active GLP-1

**Fig 5:**
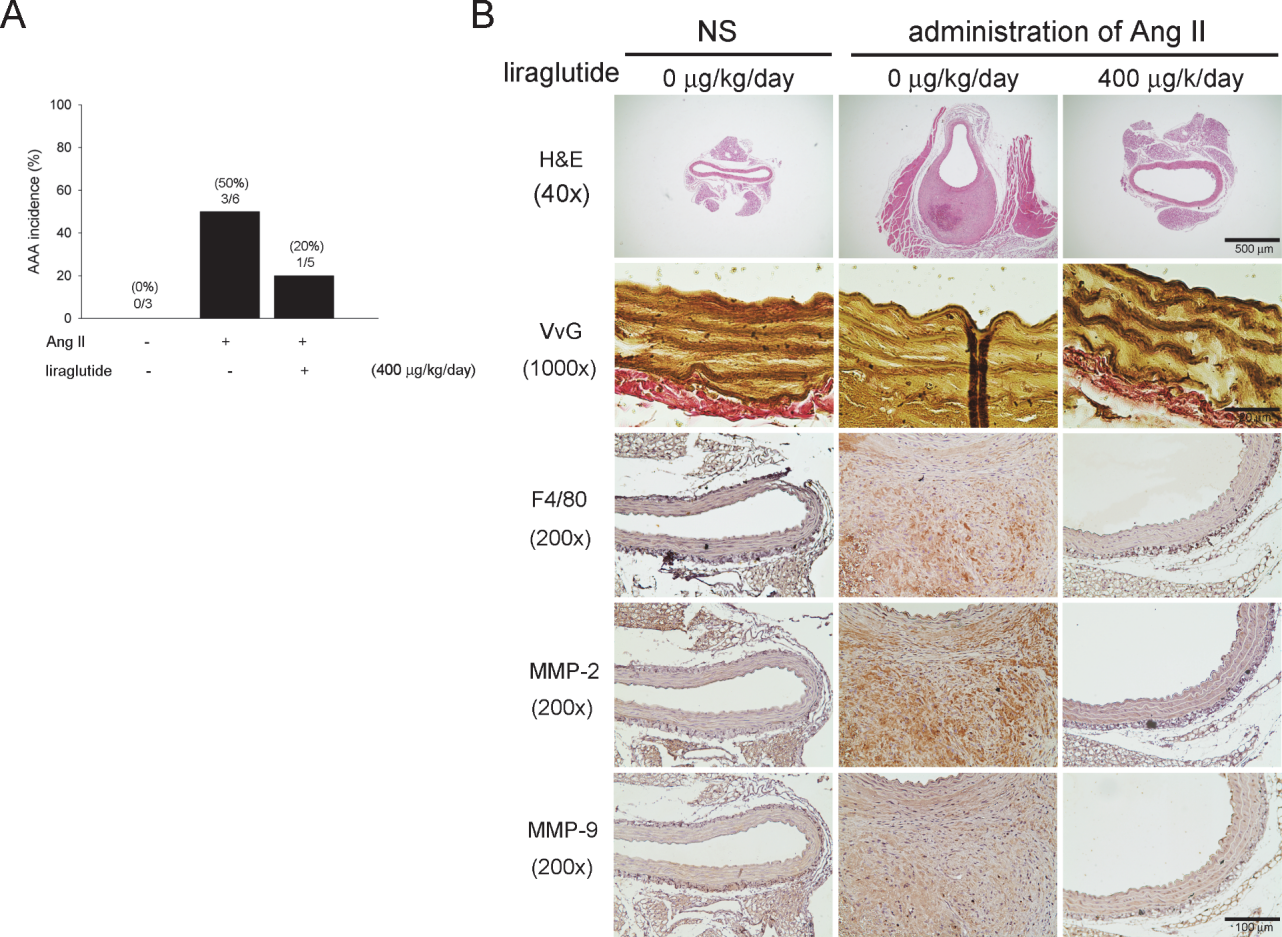
Liraglutide reduced AAA formation in Ang II-infused apoE^-/-^ mice. (A) Histogram representing the percent incidence of AAAs treated with or without liraglutide in Ang II-induced apoE^-/-^ mice. Liraglutide decreased the incidence of Ang II-induced AAA (n = 3–6 per group). (B) Representative aortic sections of the suprarenal aorta stained with H&E, VvG, F4/80, MMP-2 and MMP-9. Liragultide not only preserved elastic elements but also decreased macrophages infiltration and MMP-2 and MMP-9 expression (n = 3 per group). The magnification of immunostaining images is 200x.

Based on the observation of present study, we predicted that the benefits of sitagliptin would indirectly influence GLP-1ation to attenuate Ang II-induced AAA formation in apoE^-/-^ mice. To further clarify the probable effects of GLP-1 on AAA we tested GLP-1 analog, liraglutide, to apoE^-/-^ mice given saline or Ang II infusion. Table 3 showed the information of body weights, MAP, blood glucose and plasma of active GLP-1 level of the experimental animals. Statistical analysis showed that there were no significant differences between the three experimental groups in terms of body weight and blood glucose. Our data displayed that liraglutide did not decrease Ang II-induced MAP, however, liraglutide significantly increased the level of active GLP-1 following Ang II infusion in apoE^-/-^ mice (0.9±0.3 pM and 100.8±12.8 pM, respectively). In our experiment, to examine the influence of decreased MAP by sitagliptin we adjusted for treatment of hypertension by amlodipine in Ang II-induced apoE^-/-^mice. The results suggested that treatment with amlodipine lowered MAP in the Ang II-induced apoE^-/-^ mice, but this did not affect the extent of AAA (Data not shown).Furthermore, Animals that administrated the liraglutide group in addition to Ang II infusion showed reducing of incidence as compared with animals that infused Ang II only (20% and 50%, respectively) (Fig 5A). The diminished aortic diameters following liraglutide treatment, the maximal outer diameter, luminal diameter and wall thickness included, were measured as shown in Table 4. We also assessed the impact of liraglutide on elastin integrity, macrophage infiltration and MMP-2 and MMP-9 expression as Ang II infusion. As shown in Fig 5B, treatment with liraglutide exhibited preserving of elastin contents and decreasing of macrophages infiltration as well as MMP-2 and MMP-9 expression as compared with Ang II infused only. Hence, the result indicated that elevated active GLP-1 level suppressed AAA formation.

**Table 3:** The characteristics of liraglutide-administrated Ang II-induced apoE^-/-^ mice.

liraglutide	NS infusion	Ang II infusion	
	0 (μg/kg/day) n = 3	0 (μg/kg/day) n = 6	400 (μg/kg/b.i.d) n = 5
BW (g)	31.6 ± 3.3	31.0 ± 2.8	29.4 ± 1.8
MAP (mmHg)	82.1 ± 5.5	109.1 ± 13.6*	105.2 ± 13.3
Glucose (mg/dL)	119.0 ± 31.1	116.8 ± 30.7	115.5 ± 8.2
Active GLP-1 (pM)	1.6 ± 0.3	0.9 ± 0.3*	100.8 ± 12.8^#^

NS: Normal saline; Data are means ± SEM.

*P<0.05, compared with mice of NS infusion.

^*#*^ *P*<0.05, compared with mice of Ang II infusion with 0 μg/kg/b.i.d liraglutide administrated.

**Table 4:** The charges of aortic diameters in liraglutide-administrated Ang II-induced apoE^-/-^ mice.

liraglutide	NS infusion	Ang II infusion	
	0 (μg/kg/day) n = 3	0 (μg/kg/day) n = 6	400 (μg/kg/b.i.d) n = 5
Maximal outer diameter (% of control)	100 ± 12.1	209.6 ± 47.5*	132.6 ± 43.3^#^
Luminal diameter (% of control)	100 ± 44.4	297.2 ± 43.9*	184.9 ± 46.2^#^
Aortic wall thickness (% of control)	100 ± 13.6*	351.9 ± 65.7*	171.7 ± 113.6^#^

NS: Normal saline; Data are means ± SEM.

*P<0.05, compared with mice of NS infusion.

^*#*^ *P*<0.05, compared with mice of Ang II infusion with 0 μg/kg/b.i.d liraglutide administrated.

### GLP-1 decreased Ang II-induced ROS generation in monocytic cells

**Fig 6:**
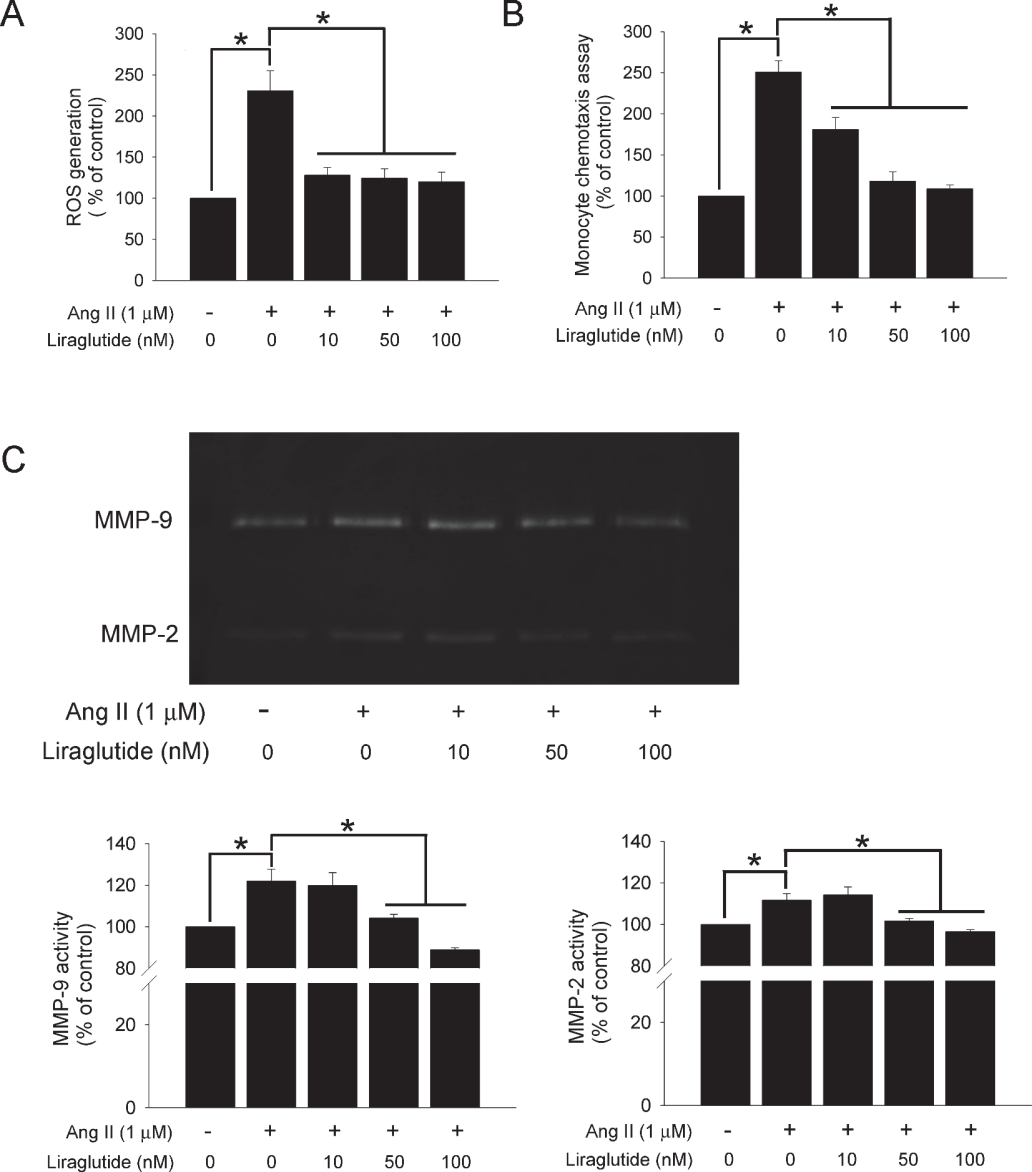
GLP-1 decreased ROS generation, migration, and MMPs activity in monocytic cells by Ang II stimulation. (A) Intracellular ROS was measured by DCF fluorescence. U937 cells were pre-treated with GLP-1 analog, liragultide, at dose of 10, 50 and 100 nM or vehicle for 1 hour, and then cells were incubated with or without Ang II (1μM) for 24 hours. (B) Chemotaxis assay was used to evaluate the effect of liragultide on U937 cell migration to fibroblast. 2x10^5^ U937 cells were treated with liragultide at a concentration of 10, 50 and 100 nM for 1 hour, and then added to the upper chamber of transwells. The lower chamber contained conditional media from adventitial fibroblast with Ang II (1μM) stimulation for 24 hours. (C) Zymography was used to determinate the gelatinolytic MMPs activity. The conditional media were obtained from the U937 cells which were pre-treated with liragultide (10, 50 and 100 nM) for 1 hour, then cells were incubated with or without Ang II (1μM) for 24 hours. (*P < 0.05, all experiment was performed in triplicate.)

Based on the observation of our in vivo results that sitagliptin administration decreased macrophage infiltration and increased plasma levels of active GLP-1, we predicted that sitagliptin would indirectly influence GLP-1 to regulate macrophage infiltration and macrophage-derived ROS production in AAAs. First, to further explore whether GLP-1 influenced ROS generation in monocytic cells, we analyzed ROS production in liraglutide-treated U937 cells that were stimulated with Ang II. U937 cells were pre-treated with liraglutide for 1 hour and then treated with Ang II (1 μM) for 24 hours. The cellular ability to produce ROS was measured by DCF fluorescence. The result showed that Ang II-treated U937 cells had greatly increased ROS production compared with control (230.9 ± 24.3% of control). However, this enhancement was decreased when cells were incubated with different doses of liraglutide (128.1 ± 9.2%, 124.3 ± 11.2%,120.1 ± 11.5%, respectively) (Fig 6A). The evidence indicated that liraglutide, GLP-1anlog, decreases Ang II-induced ROS generation in U937 cells.

### GLP-1 decreased Ang II-induced monocytes migration and gelatinase activation

Previous studies suggested that leukocyte-fibroblast interactions in the aortic adventitia potentiate IL-6 production-mediated vascular inflammation, leading to aortic aneurysm and dissection [20]. To further elucidate whether the observed reduction in adventitial macrophage content in sitagliptin-treated mice might be caused by indirect effects of GLP-1 on monocyte migration, we performed in vitro migration experiments. Because macrophages are mostly recruited to the adventitia, a migration assay was performed using fibroblast cultured conditional media by a transwell system. U937 cells were pre-treated with liraglutide at a concentration of 5–100 nM for 1 hour, and then added to the upper chamber of the transwells. As shown in Fig 6B, Ang II-stimulated adventitial fibroblasts media recruited more U937 cells than did the control (250.8 ± 13.7% of control), and this recruitment was almost entirely abolished by pre-treatment of liraglutide in a concentration-dependent manner. In addition, the evidence suggested that Ang II can also activate other ECM-degrading proteinases, such as MMP-3, MMP-9, MMP12, and MMP-13, that may contribute to the regulation of leukocyte migration [21].To investigate the effects of liraglutide on MMPs content, we tested gelatinase activity in treated U937 cells culture supernatant using zymography. Incubation with Ang II significantly increased MMP-2 and MMP-9 activity compared with control (111.7 ± 3.0% and 122.0 ± 5.7% of control, respectively). However, liraglutide inhibited MMP-2 and MMP-9 activity in Ang II-treated U937 cells (Fig 6C). The results indicate that the decrease in U937 cell migration and MMPs activity might be caused by the effects of GLP-1.

## Discussion

The present results demonstrate that treatment of sitagliptin exerts important protective effects against Ang II-induced AAA formation in apoE^-/-^ mice. We showed that sitagliptin significantly reduced the incidence of AAA in apoE^-/-^ mice infused with Ang II. Treatment with sitaglipin greatly reduced the luminal and outer diameter of the aorta. The reduction in aortic diameter associated from sitagliptin treatment was, at least in part, due to a diminution in the extent of macrophage infiltration and MMPs activity. Biochemical measurements showed that **s**itagliptin-treated mice had elevated plasma active GLP-1 level. In vitro studies demonstrated that GLP-1 results in decreased ROS generation, migration and MMPs activity in monocytic cells.

Angiotensin, a peptide hormone, gives raise to vasoconstriction and hypertension. Nevertheless, increased blood pressure do not relate to the development of AAA as interventions have been reported to eradicate AAA development unconnected of the effects on blood pressure [22]. A systemic review reported that DPP-4 inhibitor has an advantageous effect on total cholesterol and triglyceride levels, whereas its effect on the other lipids, including HDL and LDL, has not been proved [23]. Reportedly have been indicated that treatment with anagliptin significantly decreased the total cholesterol level, notably VLDL and LDL, without affecting triglyceride level in apoE^-/-^ mice [24]. However, some studies have shown that sitagliptin has no effect on triglyceride, total cholesterol, LDL or HDL levels by high fat diet induction in apoE^-/-^ mice [25,26]. Therefore, the determinate impact of DPP-4 inhibitors on the blood lipid profiles in mice remains controversial.

The maintenance-integrity structure of elastin lamina and collagen in tissues including arteries disrupted by infiltrated macrophages which secreted ECM-degradation factors, the identification of MMP-2 and MMP-9, in the aneurismal wall in both human and Ang II-infused mice AAA formation and progression have been confirmed [27,28]. Previous studies using this model demonstrated that inhibition of ECM-degrading enzymes by broad-spectrum MMP inhibitor, doxycycline, significantly decreased the incidence of AAAs [29], recommending that inhibitors of MMP activation and expression may have therapeutic benefit in AAA. Despite the secretion diverse MMPs from the monocyte/macrophage in large quantities [30], aortic smooth muscle cells increased MMP-9 expression and activity after inducible nitric oxide synthase (iNOS) inhibition by increasing NF-κB and Activator Protein-1(AP-1) activity [31]. We have now demonstrated that sitagliptin dramatically abrogates macrophages infiltration by the collaborating MMP-2 and MMP-9 expression as well as activity in aortic adventitia of the Ang II-infused mouse. In addition, the activation of MMPs is tightly regulated by TIMPs, and we have also assessed the effect of sitagliptin on TIMPs expression in Ang II-infused mouse of AAAs. The result showed that there were no significant differences between groups in our experiment (S1 Fig). This result implied that the inhibited activity of MMPs may through other intracellular pathway, or even prohibited macrophages infiltration of sitagliptin limited both expression of MMPs and TIMPs.

An earlier study illustrated the enzyme of DPP-4 has a number of substrates including other gastrointestinal hormones, neuropeptides, cytokines, and chemokines [32]. In this regard, it was important for us to demonstrate the mechanism by which sitagliptin ameliorated Ang II-induced AAA in apoE^-/-^ mice. Our study demonstrated that plasma SDF-1α levels have not significant differences in mice treated daily with sitagliptin compared to mice that were not treated. However, very little is known about whether systemic SDF-1 signals are modulated by DPP-4 activity under Ang II stimulation. Okamoto *et al*. suggested that Ang II enhances epithelial-to-mesenchymal transition via the interaction between activated hepatic stellate cells and the SDF-1/CXCR4 axis in intrahepatic cholangiocarcinoma [33]. Indirect evidence indicated that angiotensin-converting enzyme and DPP-4 utilize their proteolytic action in a balanced fashion, and angiotensin-converting enzyme inhibition may apply a compensatory modulatory effect on DPP-4 [34]. Ocana *et al*. suggested that SDF-1 is involved in the recruitment of lymphocytes within the arterial wall in AAA [35], and the leukocyte population is the most abundant source of DPP-4 positive cells in peripheral blood [5]. The leukocyte population exhibits a link between the changes levels in plasma of DPP-4 levels and this enzyme. Plasma DPP-4 may prevent their local production of bioactive peptides from systemic effects [6]. In this study, treatment with sitagliptin increased the level of plasma DPP-4 concentration but decreased plasma DPP-4 activity in Ang II-induced AAA mice. As elevated active GLP-1 levels of inhibited DPP-4 activity were also observed. Although it was unclear the vascular protectant effects of DPP-4 inhibitor from the direct effects of DPP-4 or GLP-1 elevation, previous studies have implied that GLP-1 analogues might have anti-inflammatory properties in which exendin-4 suppressed NF-κB activation in macrophages [36–38]. Various cells involve chronic inflammation in the aortic wall, chief among which, the adventitia is assumed to have an adverse role in AAA. We agreed that the GLP-1 involved in macrophage in adventitia but not fibroblast instead, despite fibroblast predominately presenting in adventitia.

Numbers of investigators have exhibited the potential-distant- strategized AAA treatments, such as the inhibition of MMPs, Ang II synthesis and receptors, the inflammatory response, oxidative stress and of the up-regulation of ECM. Although the DPP-4 inhibitor is a drug for the treatment of Type 2 diabetes, it has been reported to play a protective role in cardiovascular disease via both GLP-1 dependent and independent effects. Burgmaier et al. indicated that the effects of DPP-4 could be due to the GLP-1 action as this has shown to change MMP expression and macrophage infiltration in the aortic root [39]. With current literature’s claiming, GLP-1 analogs, such as exendin-4 and liraglutide, have shown to inhibit monocyte/macrophage infiltrator [40,41]. Bao *et al*. were the first to explore the protective effect of DPP-4 inhibitor on AAA formation. They suggested that the DPP-4 inhibitor, alogliptin, has antioxidant properties that can act as persuasive ROS scavengers in the process of AAA formation [13]. In addition Des-fluoro-sitagliptin ameliorated adipose tissue inflammation by preventing CD8^+^ T cell and M1 macrophage recruitment and decreasing plasminogen activator inhibitor-1 (PAI-1) expression in diabetic mice [42]. In 20-week-old ZDF rats fed sitagliptin (10 mg/kg) for 6 weeks, significant decreases were noted in serum levels of high sensitivity C-reactive protein and IL-1β [9].This study evidenced that oral administration of sitagliptin can prevent abdominal aortic aneurysm formation in Ang II-infused apoE^-/-^ mice, at least in part, by increasing of active GLP-1 level, decreasing of MMP-2 and MMP-9 production from infiltrated macrophage. Our results were identically concluded as previous references that DPP-4 inhibitors exert an anti-inflammatory action, suppressing the monocyte/macrophage inflammatory reaction [11,25,26,43].

The most important finding of our study, we strengthen the protective role of DPP-4 inhibitor, sitagliptin, in anti-aneurysm action by propelling the advancement of active GLP-1 with working on macrophages. Pioneering, the effect-confirmation GLP-1 analog, liraglutide, in AAA warrants our current study. Based on the observation that sitagliptin for abdominal aortic aneurysm intervention might be of significant benefits.

## Supporting Information

S1 Fig Sitagliptin did not affect TIMPs expression in Ang II-infused apoE^-/-^ mice. Representative images of TIMP-1 and TIMP-2 immunoreactivity denoted by brown staining. The expression of TIMPs were show no different among groups. Sitaglpitn were unable to increase TIMPs expression in Ang II-infused apoE^-/-^ mice (n = 3 per group). The magnification of immunostaining images is 200x.
(TIF)
