# Supplementary figures and images for: Dipeptidyl Peptidase-4 Inhibitor Decreases Abdominal Aortic Aneurysm Formation through GLP-1-Dependent Monocytic Activity in Mice
Source: PLoS One. 2015 Apr 14;10(4):e0121077. doi: 10.1371/journal.pone.0121077 (PMC4396852; doi:10.1371/journal.pone.0121077)

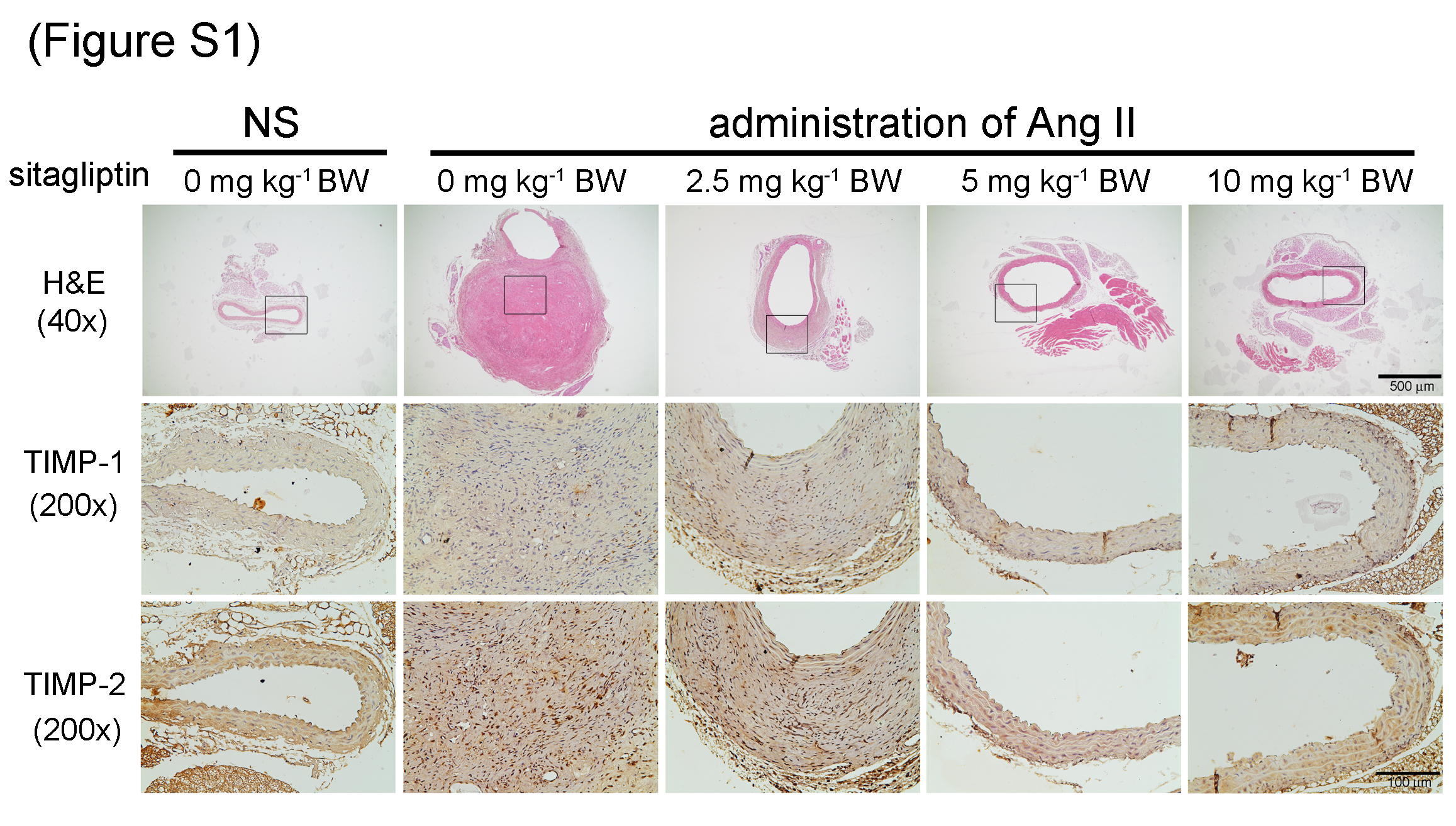

Supplement: S1 Fig — Representative images of TIMP-1 and TIMP-2 immunoreactivity denoted by brown staining. The expression of TIMPs were show no different among groups. Sitaglpitn were unable to increase TIMPs expression in Ang II-infused apoE-/- mice (n = 3 per group). The magnification of immunostaining images is 200x. (TIF) [file pone.0121077.s001.tif]
